# GC-MS Metabolomic Analysis to Reveal the Metabolites and Biological Pathways Involved in the Developmental Stages and Tissue Response of *Panax ginseng*

**DOI:** 10.3390/molecules22030496

**Published:** 2017-03-21

**Authors:** Jia Liu, Yang Liu, Yu Wang, Ann Abozeid, Yuan-Gang Zu, Xiao-Ning Zhang, Zhong-Hua Tang

**Affiliations:** 1 Key Laboratory of Plant Ecology, Northeast Forestry University, Harbin 150040, China; liujia19880906@163.com (J.L.); yangyang1990520@163.com (Y.L.); 18846034001@163.com (Y.W.); annabozeid@yahoo.com (A.A.); lpowerj@yeah.net (Y.-G.Z.); 2 Botany Department, Faculty of Science, Menoufia University, Shebin El-koom 32511, Egypt; 3 Heilongjiang Institute for Food and Drug Control, Harbin 150040, China

**Keywords:** ginsenoside, developmental stages, plant metabolism, GC-MS metabolite profiling

## Abstract

Ginsenosides, the major compounds present in ginseng, are known to have numerous physiological and pharmacological effects. The physiological processes, enzymes and genes involved in ginsenoside synthesis in *P. ginseng* have been well characterized. However, relatively little information is known about the dynamic metabolic changes that occur during ginsenoside accumulation in ginseng. To explore this topic, we isolated metabolites from different tissues at different growth stages, and identified and characterized them by using gas chromatography coupled with mass spectrometry (GC-MS). The results showed that a total of 30, 16, 20, 36 and 31 metabolites were identified and involved in different developmental stages in leaf, stem, petiole, lateral root and main root, respectively. To investigate the contribution of tissue to the biosynthesis of ginsenosides, we examined the metabolic changes of leaf, stem, petiole, lateral root and main root during five development stages: 1-, 2-, 3-, 4- and 5-years. The score plots of partial least squares-discriminate analysis (PLS-DA) showed clear discrimination between growth stages and tissue samples. Kyoto Encyclopedia of Genes and Genomes (KEGG) pathway analysis in the same tissue at different growth stages indicated profound biochemical changes in several pathways, including carbohydrate metabolism and pentose phosphate metabolism, in addition, the tissues displayed significant variations in amino acid metabolism, sugar metabolism and energy metabolism. These results should facilitate further dissection of the metabolic flux regulation of ginsenoside accumulation in different developmental stages or different tissues of ginseng.

## 1. Introduction

The metabolic network in plants is by far more extensive than those found in most other organisms. Apart from producing primary metabolites (PMs) plants also synthesize a vast range of secondary (or specialized) metabolites (SMs) [1]. PMs include the universal and essential building blocks of sugars, amino acids, nucleotides, lipids and energy sources [2,3]. The major SM classes produced by plants can be divided into three main groups: phenolic compounds, terpenoids/isopreoids, and nitrogen or sulfur containing compounds, which are produced from pathways of different PMs, including glycolysis, the TCA cycle, aliphatic amino acids, pentose phosphate pathway, shikimate pathway and notably the aromatic amino acids (AAAs) [4,5]. The tight link between metabolic fluxes of PMs and the accumulation of SMs renders the engineering of the latter compounds quite complex as it demands the consideration of the entire metabolic network in order to redirect PM resources into SMs without interfering with plant fitness [6,7]. Resource availability, for example the content of metabolically linked carbon and nitrogen compounds, has a major quantitative impact on the extent of production of SMs in plants [8,9]. Taking all of the above into consideration, metabolic engineering to stimulate the levels of these active molecules in plants is nowadays a major challenge for plant biotechnology.

*Panax ginseng* C.A. Meyer, a traditional medicinal herb, has been used for thousands of years. It offers several types of therapeutic benefits [10,11], including anti-stress, health promotion, maintenance and enhancement of the immune systems, anti-oxidation, prevention of certain chronic diseases and aging deterrence properties. Ginseng’s bioactive elements, including ginsenosides, phenols, and essential oils, have turned out to be effective as anticancer agents [12,13,14,15]. Additionally, ginseng tissues have been reported to contain amino acids, fatty acids, tritepenes saponins, poyacetylenes, alkaloids, polysaccharides, vitamins and phenolic compounds, with its biochemical and pharmacological activities being valuable and expansive [16,17]. Previous studies have suggested that these bioactive components correlate with the age and tissue of ginseng [18]. Years-old ginseng is generally more expensive than younger ginseng products because older ones are often selected for further processing and are recognized as being of higher quality due to its efficacy and external shape.

Metabolomics is an omic approach that aims at the systematic study of endogenous small molecule metabolites [19,20,21]. Metabolites are the end products and intermediates of various metabolic pathways, thus any changes in pathology or physiology can be reflected in the metabolic profiling [22,23]. To date, metabolomics has been a promising analytical technology that has been used to unravel the metabolic fluctuations of crucial plant molecules and the metabolic fluxes associated with various research areas, especially in growth stages and tissue compound discovery. For example, comprehensive profiling and the natural variation of flavonoids in rice, analysis of medicinal *Panax* herbs, and ginseng cultivation age determination were all studied using metabolomics strategies [24,25,26,27,28]. Gas chromatography-mass spectrometry (GC-MS) has also been widely employed in metabolomics studies, due to the high quality and reproducibility of the data, wide dynamic range, availability of universal mass spectral libraries and the ability to detect hydrophilic metabolites after derivatization [17,26,29,30]. This approach integrates the advantages of targeted and untargeted methods and has been used to study the metabolic profiles of various tissues samples, for instance, tissue effects of carbon sources on metabolites were tested in plants after leaf detachment, phloem-peeling, and silique-darkening, and to study the dynamic changes of metabolic responses to geographical fluctuations during tobacco growth stages [29,31]. The metabolite profiling of *Panax* herbs including Chinese ginseng, American ginseng and Korean ginseng has been carried out successfully with the developed UPLS-TOFMS method and multivariate statistical analysis [32]. Yang et al. cultivated ginseng root samples in a restricted and controlled area according to standardized cultivation protocols, and then the ginseng samples were analyzed by two-dimensional NMR-based metabolomics techniques using various solvents to develop a differentiation method for ginseng cultivation age [33]. In addition, several studies concerning the responses of ginsenoside biosynthesis to environmental conditions and growth stages have been conducted in ginseng plants [18,34], yet relatively few studies have focused on the comprehensive profiling of primary metabolites responses to tissue and developmental stage differences.

A complete understanding of the dynamic changes in crucial intermediates which affect total ginsenoside accumulation is required to elucidate the influence of primary metabolites during the different plant growth phases and within different tissues in *P. ginseng*. To this end, this study first examined the dynamic changes of metabolites to generate a global picture of the metabolites present at different stages within different tissues during *P. ginseng* development. Primary metabolites which are associated with ginsenoside accumulation and may be potential targets for enhancing the ginsenoside content in *P. ginseng* were tested in different tissues within plants at different growth stages (1-, 2-, 3-, 4-, 5-years) using GC-MS analysis combined with multivariate statistical analysis. Taken these together, our findings help improve our understanding of the biochemical processes and tissues responsible for ginsenosides accumulation, leading to the development of methods for increasing the content and quality of ginsenosides in *P. ginseng*.

## 2. Results

### 2.1. Total Ginsenosides Accumulation in Different Tissues during Different Developmental Stages

**Figure 1:**
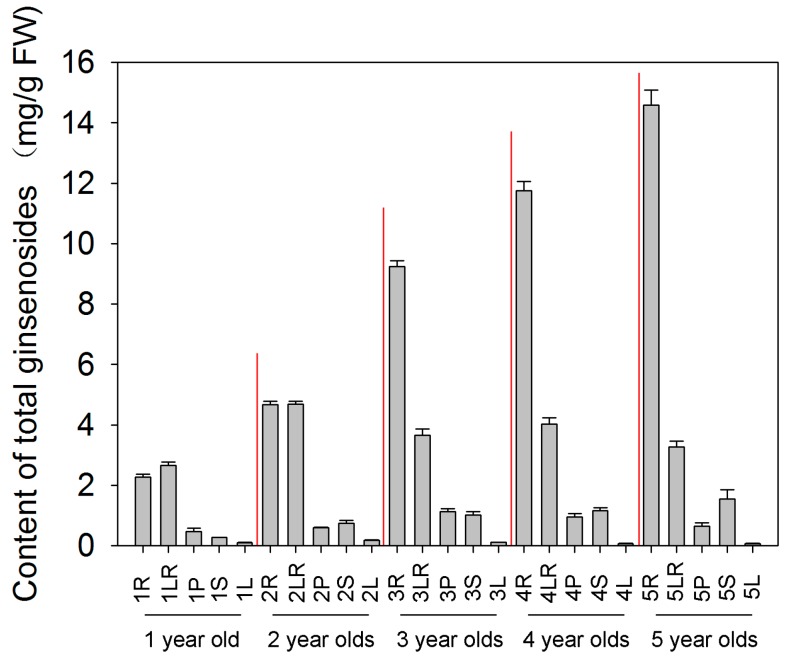
Total ginsenosides analysis of *ginseng* at five developmental stages and five tissues. Ginseng samples of five developmental stages 1-, 2-, 3-, 4- and 5-year old were collected for ginsenosides analysis by UPLC-MS. The data represent the mean ± standard error of the mean values for six biological replications.

Initially, we developed a UPLC-MS method for the simultaneous quantification of ten ginsenosides, including Rg1, Re, Rd, Rb1, Rb2, Rf, Rh, Rh2, Rg3 and Rc (Table S1). Moreover, this study showed the total content of these ten ginsenosides in different tissues during different developmental stages (Figure 1). For most developmental stages, the total ginsenosides showed a similar trend, and mainly accumulated in roots, followed by lateral roots (Figure 1). However, a small amount exists in leaves. In addition, the range of total ginsenosides content accumulated in the underground tissues, including root and lateral roots, was two to eight times greater than its range in the above-ground tissues, including leaves, petioles and stems, in the young ginseng. Surprisingly, the underground tissues range reached sixteen to twenty-two times greater levels than the aboveground tissues range in the old ginseng. This supports the recent trend towards the usage of the roots in medicine.

In Figure 1, the total content of ginsenosides increased with ginseng age in roots and stems. However, in lateral roots and leaves, it was not difficult to determine that the ginsenoside content increased sharply in the second year, while the content of old ginseng was two times lower than that in the young ginseng. In addition, there was a sharp increase in petiole content during the first three years, followed by a gradually decrease during the fourth and fifth year. These results indicated that the correlation between the developmental stage and ginsenoside content in the same tissue is complex, and may be regulated by the synthesis of other metabolites.

### 2.2. Metabolic Profiling of Cultivated Ginseng during Developmental Processes

*Panax ginseng* is a perennial herb that grows in the spring and sheds its leaves by October to prepare the roots for winter. In ginseng the stalk of the plant changes every year until the 5th spring. The shoot dies every year in the fall and creates a death scar. The older the plant the more scarred it becomes. The plant takes 9–10 months to remove the aboveground tissues, followed by 6–7 months to re-grow the stem, petiole and leaf. To investigate the global metabolic changes of ginseng in response to plant growth periods; green leaves, stems, petioles, lateral roots and roots were collected from JiLin. And 1-year, 2-year, 3-year, 4-year and 5-year-old plants were studied.

**Figure 2:**
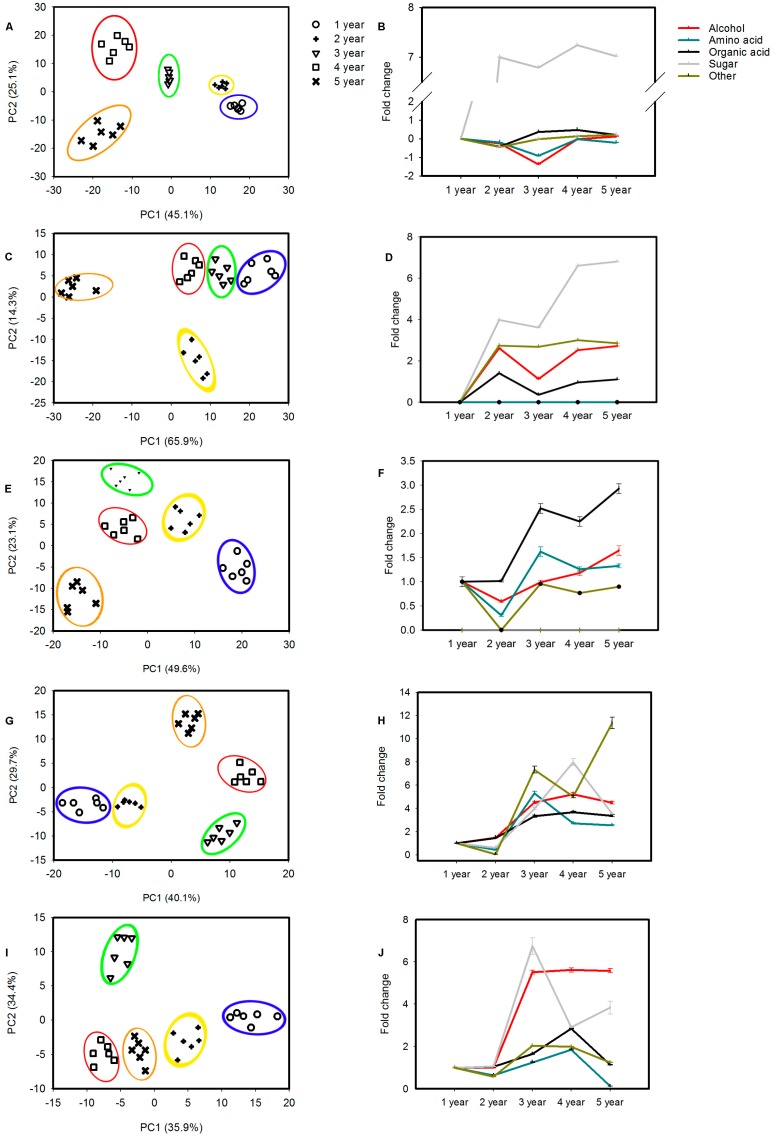
Metabolic analysis of tissues at five developmental stages. *Ginseng* samples of five developmental stages 1-, 2-, 3-, 4- and 5-year old were collected for metabolite analysis by GC-MS. (**A**,**C**,**E**,**G**,**I**) PLS-DA analysis of developing leaf, petiole, stem, lateral root and root from five stages, respectively; (**B**,**D**,**F**,**H**,**J**) Fold change of alcohol, amino acid, organic acid, sugar and other metabolites in leaf, petiole, stem, lateral root and root at five developmental stages. The data represent the mean ± standard error of the mean values for six biological replications.

A supervised PLS-DA analysis, which could classify the observations into the group from giving the largest predicted indicator variable, with unit variance (UV) scaling (variables-subtracted average value divided by the standard deviation) was conducted to visualize the effects of growth stages on metabolites (Figure 2A,C,E,G,I). Each model parameter (R2X = 0.885, Q2 (cum) = 0.727; R2X = 0.879, Q2 (cum) = 0.707; R2X = 0.92, Q2 (cum) = 0.717; R2X = 0.888, Q2 (cum) = 0.728; R2X = 0.907, Q2 (cum) = 0.716) indicated that 88.5% and 72.7%, 87.9% and 70.7%, 92% and 71.7%, 88.8% and 72.8%, and 90.7% and 71.6% of the total variations are explained and predicted in the same tissues’ different developmental processes, respectively. Figure 2 shows the resulting score plots derived from the optimal PLS-DA model of ginseng samples according to cultivation age. Samples of 1-year, 2-year, 3-year, 4-year and 5-year-old were separated in five different tissues.

The altered metabolites were found from the line plots of the X-loadings of the first component of the PLS-DA pairwise comparison models. It was reported that the variable importance in the projection (VIP) values greater than 1 indicated the most relevant metabolites for explaining the different growth periods. On the basis of the parameter VIP > 1, 30 leaves, 16 stems, 20 petioles, 36 lateral roots, and 31 roots-responsive metabolites with significant changes (student′s T-test *p* < 0.05), were identified, respectively (Supplementary Figure S1). The annotated metabolites were divided into five categories: amino acids, sugars, organic acids, alcohols and other (specialized metabolites). The level of metabolites in the sugars category was increased in developmental stages of leaves and petioles (Figure 2B,D) but reduced by year 4 and 3 in lateral roots and roots, respectively, although a dramatic raise of the total ginsenoside content was detected in roots (Figure 1). Surprisingly, we found an accumulation of organic acids in stems and lateral roots. Consistent with that, the levels of ginsenosides accumulate in the growth process of the stems. Interestingly, the patterns of change in alcohol levels were similar to those of total ginsenosides in roots and stems (Figure 1). Consistent with this finding, organic acids were significantly increased in lateral roots. However, lower level of sugars and amino acids were identified in 1–5 year olds stems and petioles, respectively (Figure 2D,F), further indicating that the different tissues affected the metabolite influx during the total ginsenoside accumulation in different plant growth stages. In addition, the level of other specialized metabolites was significantly increased in petioles of ginseng (Figure 2F), although a significant accumulation of ginsenosides in petioles was only found in 3 year old plants (Figure 1). These results suggest some discrepancies between the contents of primary metabolites and ginsenosides during plant growth.

### 2.3. Tissue Accumulation of Metabolites in Various Tissues of Ginseng

**Figure 3:**
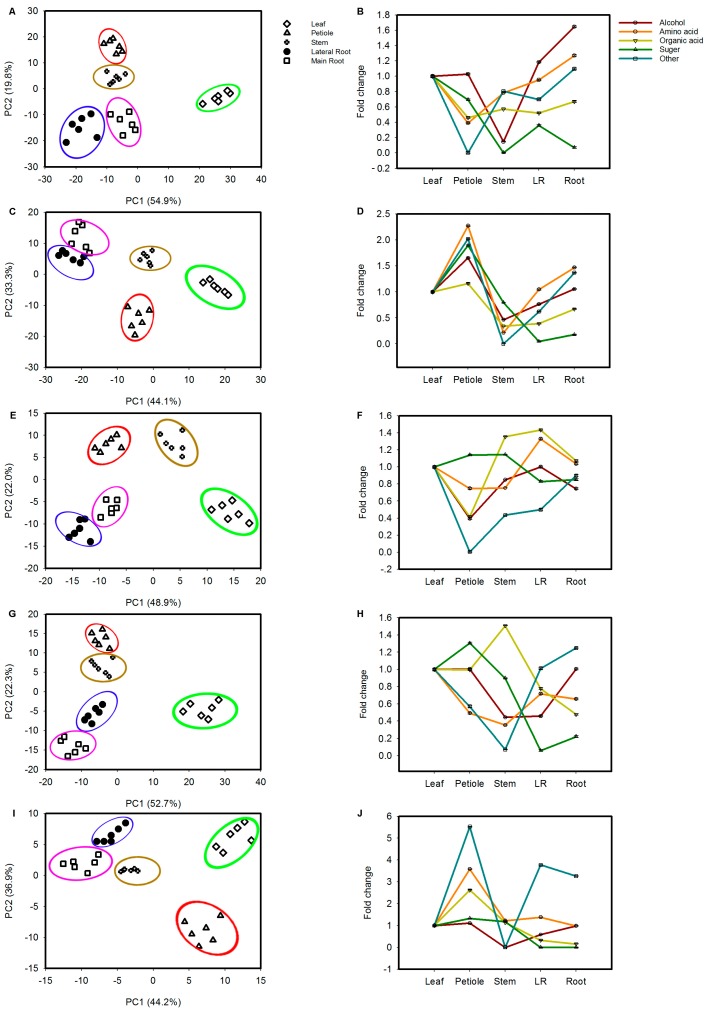
Metabolic alterations in five tissues during developmental. *Ginseng* samples of five developmental stages 1-, 2-, 3-, 4- and 5-year old were collected for metabolite analysis by GC-MS. (**A**,**C**,**E**,**G**,**I**) PLS-DA analysis of 1-, 2-, 3-, 4- and 5-year from leaf, petiole, stem, lateral root and root, respectively; (**B**,**D**,**F**,**H**,**J**) Fold change of alcohol, amino acid, organic acid, sugar and other metabolites in 1-, 2-, 3-, 4- and 5-year at five tissues. The data represent the mean ± standard error of the mean values for six biological replications.

To clarify the metabolic characteristics associated with the same growth stages, responses to different ginseng tissues, including leaves, stems, petioles, lateral roots and roots during the 1~5 year developmental process were investigated. Figure 3 shows that the PLS-DA results allowed for good discrimination among samples.

In the PLS-DA pattern, each mode parameter (R2X = 0.902, Q2 (cum) = 0.722; R2X = 0.917, Q2 (cum) = 0.73; R2X = 0.876, Q2 (cum) = 0.708; R2X = 0.883, Q2 (cum) = 0.696; R2X = 0.938, Q2 (cum) = 0.727) indicated that 90.2% and 72.2%, 91.7% and 73%, 87.6% and 70.8%, 88.3% and 69.6%, and 93.8% and 72.7% of the total variations are explained and predicted in the same tissues’ developmental processes in different tissues, respectively.

To investigate the change in alcohols, amino acids, organic acids, sugars and specialized metabolites in five tissues of the same developmental stage; for each tissue, a mixture of samples was obtained (Supplementary Figure S2). To find the potential compounds, a parameter VIP > 1 test was performed to define the variables associated with tissues. Totals of 24, 29, 33, 25 and 14 metabolites (*p* < 0.05) were markedly altered following tissue changes, respectively (Figure S2). In the first year, roots contained the highest levels of alcohols, amino acids and other metabolites, followed by lateral roots, petioles, leaves and stems. However, compared with leaves, other tissues showed the lowest accumulation of sugars and organic acids. In the second year, petioles contained the highest levels of the five categories, followed by leaves and stems; they all belong to the aboveground tissues. In subsequent years, the highest levels of sugars and other metabolites showed similar trends in aboveground tissues, including (leaves, followed by stems and petioles). In addition, the highest levels of amino acids and other metabolites were significantly accumulated in the fourth year in leaves, followed by petioles and stems. Interestingly, sugars were increased in leaves, followed by petioles, stems, lateral roots and roots, despite the opposite trend in ginsenoside accumulation in the fifth year. Taken together, our results suggest that primary metabolites biosynthesis is regulated in a tissue-dependent manner.

### 2.4. Pathway Mapping and the Metabolite-to-Metabolite Network Visualization

**Figure 4:**
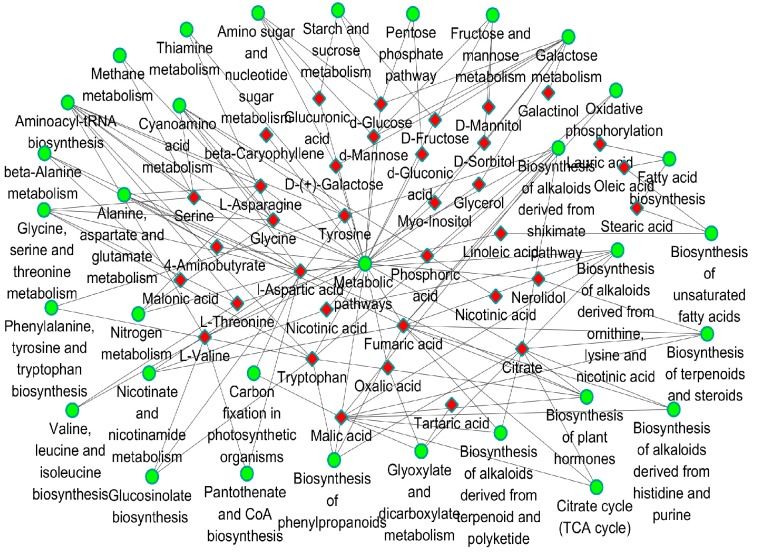
The metabolite-to-metabolite networks involved in the primary pathways of ginseng response to development regulation. *Green circles,* metabolite pathways; *Red diamonds*, metabolites.

All of the changed metabolites affected by developmental processes and tissues were mapped to the biological pathways involved in the KEGG database, which were assigned to 62 and 41 pathways in physiological stages and tissues, respectively (Supplementary Tables S3 and S4). The most statistically enriched pathways were analyzed by a Bonferroni correction (*p* < 0.05). The results showed that 24 pathways were enriched with changed metabolites, as a result of stages and tissues, respectively (Supplementary Table S5). Among them, 13, including biosynthesis of alkaloids derived from histidine and purine, biosynthesis of alkaloids derived from ornithine, lysine and nicotinic acid, biosynthesis of alkaloids derived from shikimate pathway, biosynthesis of alkaloids derived from terpenoid and polyketide, biosynthesis of phenylpropanoids, biosynthesis of plant hormones, biosynthesis of terpenoids and steroids, citrate cycle (TCA cycle), fatty acid biosynthesis, fructose and mannose metabolism, galactose metabolism, and glyoxylate and dicarboxylate metabolism were enriched for both the stages and tissues-regulated metabolites. Furthermore, using all the altered metabolites and pathways as inputs, we constructed metabolite-to-metabolite interaction networks comprising 34 metabolites for the stages and tissues-regulated in ginseng, respectively (Figure 4). Both networks could be divided into two sub-clusters. One sub-cluster consisted of compounds mainly involved in carbohydrate and glycan biosynthesis, and the other was composed of several kinds of acidic compounds, including amino acids and organic acids.

## 3. Discussion

Ginsenosides are the most important pharmaceutically active compounds in ginseng herb. Extensive studies confirm that the total ginsenosides content increases year by year in roots [35]. In this study, we present the first detailed study of metabolic changes occurring during the accumulation of ginsenosides in the 1-, 2-, 3-, 4-, and 5-year-old stages of ginseng within five different types of tissue (roots, lateral roots, petioles, stems, and leaves). We found a series of metabolites, including derivatives of sugars, amino acids, organic acids and other compounds (Table S6). The abundances of these components undergo dynamic changes and have a significant correlation with ginsenoside accumulation within the different plant tissues during different developmental stages (Table S7). The understanding of the patterns of the changes in metabolite composition during the developmental stages and tissue-dependent metabolic shifts observed in this study may shed new light on the enhancement of ginsenoside levels in developing plants.

Four-six years before harvest, the plant becomes lignified when it is more than 6 years old, thus decreasing its merchantable quality [36], and in consequence, plant development can be divided into five stages corresponding to 1-, 2-, 3-, 4-, and 5-year-old plants. During these stages, various biochemical processes and relevant metabolites undergo dynamic metabolic changes [37,38]. Therefore, elucidating the metabolomics dynamics associated with progression to harvest could aid in the identification of previously undescribed compounds that may be useful for enhancing ginsenoside content in ginseng. Our time-dependent profiling of metabolites content in developing tissues revealed a rapid shift in metabolites at the 1-, 2-, 3-, 4-, and 5-year-old stages in each tissue (Figure 2). Carbohydrates, including glucose, fructose and sucrose, are the main precursors of secondary metabolite biosynthesis in developing plants [39], and in our metabolites profile d-fructose, melezitose, ribose, mannose and d-(−)-tagatose showed considerable increases in the leaf followed by petiole after the first year of growth, while they showed slight increase and decrease in root and lateral roots, respectively. This was expected as a result of photosynthesis in the aboveground green parts. However, after the second year of growth, this observation was reversed with an unexpected decrease in carbohydrates in the photosynthetic parts and a considerable increase in the underground part accompanied by rapid accumulation of ginsenosides in roots, which leads us to believe that carbohydrates that were synthesized in the aboveground part during the first two years were transferred after the second year to the underground parts resulting in the unexpected increase in carbohydrates in roots and lateral roots. This metabolic shift to the underground parts could play a role in ginsenoside biosynthesis in underground parts. Actually after the third and fourth years of growth carbohydrates decreased gradually in roots and lateral roots, respectively, which could be due to sugar consumption in the ginsenoside biosynthesis process. In conclusion, high concentrations of metabolites transferred from aboveground tissues to regulate the metabolic flux in underground tissues, eventually leading to enhanced ginsenoside content in roots and lateral roots.

The production of secondary metabolites showed a gradual accumulation in developing *P. ginseng* plants, as was also discovered in our study. They almost accumulated in underground parts (main- and lateral roots) with the growth stage, follow by petioles, stems and leaves.

The total organic acids content was dramatically increased after the second year in leaves, for example, a 1.5- and 2-fold increase in nicotinic acid and lauric acid were observed, respectively, while 4-aminobutyrate was reduced by approximately 3-fold (Figure S1E). However, the contents of ginsenosides showed an opposite trend, which leads us to infer that the organic acids would be utilized for ginsenoside biosynthesis in the leaves. The level of citrate and oxalic acid showed significant accumulations in main root and lateral root after the second year (Figure S1A,B), and ginsenosides were accumulated more there than other tissues at 2~5 years, suggesting that organic acids would be positive regulators for ginsenoide biosynthesis in main roots and later roots. The organic acids content increased significantly during the plant growth process in stems, for example, *cis*-sinapic acid, malic acid, decanedioic acid and lauric acid (Figure S1D). These metabolites could significantly promote the synthesis of total ginsenosides.

Amino acids, as the biosynthetic precursors of some antioxidants, are upregulated to provide some osmoprotective functions in plants [40,41]. The levels of most amino acids (e.g., tyrosine, dl-glutamine, l-valine, tryptophan, dl-glutamine, glycine and isoleucine) were lower after two-years, and consumption of these compounds likely causes ginsenoside synthesis to be blocked in underground tissues (main and lateral roots) (Figure S1A,B). However, some other amino acid (isoleucine, kynurenine, serine and lysine) were higher after the second year at aboveground tissues (stem and leaf). Amino acids have different accumulation trends between under- and aboveground plant parts, and have a reverse trend with ginsenoside biosynthesis, as these indicated amino acids were accumulated to promote the biosynthesis of ginsenosides in the underground parts, while these primary compounds were consumed to increase the accumulation of ginsenosides in aboveground parts.

Additionally, increased levels of sugar alcohols (e.g., glycerol, galactinol, d-allose and epiglobulol) derived from the pentose phosphate (PPP) pathway were observed during plant growth in roots (Figure S1A) and the same trend as ginsenoside biosynthesis, likely reflecting the higher respiratory rate of the PPP pathway to regulate ginsenoside biosynthesis. The positive correlation between ginsenoside contents and the levels of sugar alcohols was also detected in leaves and petioles that showed decreases of sugar alcohol levels after the second year accompanied by a decrease in ginsenoside contents in the same parts after the second and the third year, respectively. These results show that the higher rate PPP metabolism and energy metabolism in ginseng provides sufficient C-skeletons and energy for the plant’s physiological metabolism and ginsenoside biosynthesis.

In most plants, leaves are the major photosynthetic tissue [27]. In ginseng, three potential tissues can act as carbon sources to enhance metabolite production in developing plants: leaves, petioles, and stems. However, there is a series of questions regarding the contributions of these three carbon sources tissues for secondary metabolites accumulation during plant growth stages. Sugar metabolism belongs to C metabolism, which is responsible for the production of accessible energy, resistance metabolites and carbon skeletons for plant life activities during growth and development, which are the degradation products of sucrose and starch [2,42]. We observed a sharp decline in sugar metabolism in leaves, followed by petioles, stems, lateral roots, and roots in different growth processes (Figure 3), despite an opposite trend in total ginsonoside biosynthesis. These results implied that higher rates of photosynthesis occurred and higher energy was consumed in aboveground parts (leaves, petioles, and stems) to regulate underground (lateral root and root) tissues’ bioactive compound biosynthesis. Additionally, for amino acids, as important energy metabolites and synthetic precursors of various bioactive molecules, the levels most were lower in lateral roots and roots (Figure S2). This seems to imply energy metabolites expenditure to enhance ginsenoside accumulation.

## 4. Materials and Methods

### 4.1. Plant Materials

*P. ginseng* samples were grown in a field belonging to the Changbai Mountain field base located in Northeast of China (42°01′–43°24′ N, 127°48′–129°11′ E). All samples grew under the same temperature, water, soil, humidity and altitude conditions. A total of 150 fresh ginseng tissues, including 30 samples from leaves, 30 samples from petioles, 30 samples from stems, 30 samples from lateral roots and 30 samples from roots during five developmental stages (1-, 2-, 3-, 4- and 5-year), were collected and investigated in this study (Supplementary Table S2). All samples were collected at approximately ten o’clock in the morning to avoid the effects of diurnal variations on the metabolic profiles. Six biological duplicates of each sample were collected and rapidly frozen in liquid nitrogen to inhibit any decline of enzyme activity in the plant tissue. All harvested ginseng samples were washed with tap water for analysis according to cultivation age and tissue by being individually freeze-dried, then ground to a fine powder using a mortar and pestle. Powdered samples were then stored at −70 °C until GC/MS analysis.

### 4.2. Sample Preparation

In total, six biological replicates of each cultivation age and tissue group were subjected to GC/MS analysis. In brief, 60 mg of each ginseng sample was transferred to 1.5 mL tube. Metabolites were extracted by the addition of 360 μL methanol (pre-cooled at −20 °C) and 40 μL internal standard (2-l-chlorophenylalanine), followed by vortexing for 2 min, then sonication for 30 min, and after the addition of 200 μL of chloroform and 400 μL of water, sonication again for 30 min. Subsequently, the tubes were centrifuged at 14,000 rpm at 4 °C for 10 min. The supernatant was transferred to a derivatized glass bottle, and evaporated to dryness. A derivatization method (oximation) was used to increase the volatility of metabolites. The dried residue was redissolved in 80 μL methoxyamine pyridine solution (15 mg/mL) and incubated for 90 min at 37 °C. Subsequently, an 80 μL aliquot of BSTFA (including 1% TMCS) was taken and 20 μL were added to the mixtures, and after vortexing for 2 min, they were then derivatized for 60 min in a 70 °C. Prior to injection, the solution was centrifuged at 12,000 rpm for 5 min to remove precipitates, and the supernatant was transferred to a glass vial for injection. All these solutions were analyzed by GC/MS.

*Panax ginseng* tissues (1.0 g) were weighed accurately, subjected to Soxhlet extraction and extracted overnight at 4 °C with 60 mL distilled water-saturated *n*-butanol. The next day, the extract solution was filtered, the filtrate was separated with a funnel and washed with 20 mL 2% NaOH solution. The upper solution combined and evaporated to dryness, and diluted to the desired volume with methanol. Following centrifugation at 10,000 *g* for 15 min, the extracts were absorbed and filtered through a 0.45 μm syringe filter prior to UPLC analysis.

### 4.3. GC-MS and LC-MS Based Metabolomic Analysis

Each 1 μL aliquot of the derivatized solution was injected in splitless mode into an Agilent 7890A-5975C GC-MS system (Agilent Technologies, Santa Clara, CA, USA). Separation was carried out on a non-polar DB-5 capillary column (30 m × 250 μm I.D., J&W Scientific, Folsom, CA, USA), with high purity helium as the carrier gas at a constant flow rate of 1.0 mL/min. The GC temperature programming began at 60 °C, followed by 8 °C/min oven temperature ramps to 125 °C, 4 °C/min to 210 °C, 5 °C/min to 270 °C, and 10 °C /min to 305 °C, and a final 3 min maintenance at 305 °C. The electron impact (EI) ion source was held at 260 °C with a filament bias of −70 V. Full scan mode (*m*/*z* 50–600) was used, with an acquisition rate of 20 spectrum/second in the MS setting.

Chromatographic separation was performed on an Ultra-Performance LC (UPLC) system (Waters, Tokyo, Japan) with a LC-20AD pump, a temperature controller, column oven, SIL-20A autosampler (Waters), and an ACQUITY UPLC BEH C_18_ column (1.7 μm, 2.1 mm × 50 mm) with in-line filter and maintained at 25 °C was used. The mobile phase in UPLC determinations is consisted of (A) water and (B) acetonitrile. The elution program was optimized as follows: 30% B (0–4.5 min), 30%–60% B (4.5–6 min), 60%–90% B (6–6.5 min), 90% B (6.5–7.5 min), 90%–30% B (7.5–8 min), 30% B (8–10 min). The flow rate was 0.25 mL/min and the injection volume was 5 μL. Tandem mass spectrometric detection was performed on an QTRAP 5500 Ion trap mass spectrometer (AB SCIEX, Foster City, CA USA) equipped with an electrospray ionization (ESI) source in the positive ion detection mode. MS source conditions were set as follows: ion spray voltage −5500 V, turbo spray temperature –500 °C, high purity nitrogen was used in all units; nebulizer gas −25 psi; curtain gas −20 psi, positive ion mode was finally employed. The *m*/*z* was 121.050873 purine (purine, C_5_H_4_N_4_) and *m*/*z* was 922.009798 the HP-921 (hexakis-1*H*,2*H*,3*H*-terrafluoropentoxy)phosphazene, C_18_H_18_O_6_N_3_P_3_F_24_) as a precise molecular mass internal standard. Data acquisition was performed with Analyst 1.4.2 software version (AB SCIEX, Concord, ON, Canada). The optimized method was employed for the determination of the ginsenosides by comparing the area calculated for each peak to the standard curves obtained from authentic ginsenoside standards (Table S8).

### 4.4. Statistical Analysis

Raw GC/MS data were converted into CDF format (NetCDF) using the Agilent GC/MS 5975 data analysis software (version is 14.1, Agilent Technologies, Santa Clara, CA, USA) and were subsequently processed by the XCMS (www.bioconductor.org). Each metabolite was expressed as peak area normalized to the IS. For multivariate statistical analysis, the XCMS output was further processed using Microsoft Excel. Finally, normalized data were imported into SIMCA-P software (version 11.0, http://www.umetrics.com/simca)for multivariate statistical analysis, which used to partial least squares discriminant analysis (PLS-DA). All data were mean-centered and UV-scaled prior to PLS-DA. Cross-validation was used to calculate the number of significant components. Permutation test was used to estimate the validity of PLS-DA model, 999 times of permutation was used in all models. For a valid model, R2 intercept is less than 0.4, and Q2 intercept is less than 0.05, otherwise the model is considered as overfitted. Discriminating metabolites were identified using a statistically significant threshold of variable influence on projection (variable influence on projection values, VIP > 1.0) values obtained from the PLS-DA model and were further validated by *t*-test analysis. Metabolites with VIP values greater than 1.0 and *p* values below 0.05 (threshold) were selected as discriminating metabolites between different classes of samples. A heat map analysis was conducted with R (www.r-project.org/) to visualize the relationships and relative levels of metabolites. The correlation significance and Duncan’s honestly significant difference (HSD) *post-hoc* test were calculated using the SPSS software (version 17.0, SPSS, Santa Clara, CA, USA), and the metabolic correlation network with factors was constructed using the Cytoscape V2.8.3 software [43].

## 5. Conclusions

*P. ginseng* is a major herbal plant. Understanding the dynamic changes of its growth and tissues metabolic flux is a critical step to increase the quantity and quality of ginsenosides, its major bioactive components. We profiled metabolites in plant tissues of different developmental stages that underlie the ginsenoside accumulation process and identified several differentially expressed metabolites associated with total ginsenoside accumulation. Interestingly, metabolic profiling of different plant tissues during different developmental stages showed that high concentrations of metabolites (e.g., d-fructose, d-(+)-galactose, and d-turanose) transferred from aboveground tissues to regulate underground tissues’ metabolic flux, eventually leading to enhanced ginsenoside content in roots and lateral roots. Moreover, we could infer that the primary metabolite alcohols (e.g., glycerol, galactinol, d-allose and epiglobulol) may play a critical role in ginsenoside accumulation in roots. These findings may provide a physiological and agronomical strategy database for the more efficient synthesis of ginsenosides in *P. ginseng*.

## Supplementary Materials

The supplementary materials are available online.
